# Pregnancy and Neonatal Outcomes for Women Without Male Partners Undergoing Fertility Care via Intrauterine Insemination: A Retrospective Cohort Study

**DOI:** 10.3390/jpm15120589

**Published:** 2025-12-02

**Authors:** Wendy Y. Zhang, Megan McCracken, Amy Zhang, Lisandra Veliz Dominguez, Lusine Aghajanova

**Affiliations:** 1 Department of Obstetrics and Gynecology, Stanford University School of Medicine, Stanford, CA 94087, USA; 2 Division of Reproductive Endocrinology and Infertility, Department of Obstetrics and Gynecology, Stanford University School of Medicine, Sunnyvale, CA 94087, USA; 3 Quantitative Sciences Unit, Department of Medicine, Stanford University School of Medicine, Stanford, CA 94304, USA; 4 Department of Pediatrics, Stanford University School of Medicine, Stanford, CA 94087, USA

**Keywords:** lesbian, single women, intrauterine insemination, infertility, pregnancy outcomes, neonatal outcomes

## Abstract

**Objective:** The objective of this study is to examine the detailed pregnancy and neonatal outcomes of women without male partners undergoing intrauterine insemination (IUI) compared to women with male partners. **Methods:** This is a retrospective cohort study of all patients who completed an IUI cycle from 2017 to 2023. 2414 cycles were included in the study: 149 cycles for women without male partners (including single and lesbian women) and 2265 cycles for women with male partners. Primary outcomes were the rates of clinical pregnancy, miscarriage, and live birth. Secondary outcomes were obstetric complication rates and neonatal outcomes. **Results**: Women without male partners undergoing IUI were significantly older than the reference cohort (median age 42 years versus 38 years, *p* < 0.0001). 84.1% of women without male partners did not have a diagnosis of the common causes of female infertility. Both cohorts had similar cycle characteristics and number of IUI cycles until pregnancy and live birth. The mean clinical pregnancy rate per cycle for women without male partners was 11.4% versus 12.5% for the reference group (*p* = 0.56), and the mean live birth rate was 8.1% versus 8.2% (*p* = 0.95). Multiple pregnancy, cumulative pregnancy, and clinical miscarriage rates were also similar. Similarities persisted after adjusting for confounders: age, BMI, race, and infertility diagnosis. Importantly, there were no statistically significant differences in obstetric complications (such as hypertensive disorders of pregnancy, gestational diabetes, placental disorders) and neonatal outcomes. **Conclusions**: Compared to women with male partners undergoing IUI, women without male partners had similar rates of clinical pregnancy (per cycle and cumulative), miscarriage, and live birth; there were no significant differences in obstetric complications or neonatal outcomes.

## 1. Introduction

As the utilization of assisted reproductive technology grows, there has been increasing diversity in the patient population accessing fertility services [1]. However, the vast majority of outcome data used to guide patient counseling and treatment has largely been based upon studies of partnered, cisgender heterosexual couples. Patients who do not conform to this identity not only face barriers to accessing fertility care but are also underrepresented in the scientific literature [2,3,4]. As such, the American College of Obstetrics and Gynecology encourages providers to ensure that “clinical spaces are affirming and open to all patients, such that equitable and comprehensive reproductive health care can meet the needs of these communities” [5]. Furthermore, the 2021 American Society for Reproductive Medicine committee opinion highlighted that access to reproductive technology should be equal “without regard to marital status, sexual orientation, or gender identity” [6]. A component of that comprehensive and equitable care should entail inclusion in scientific studies that guide evidence-based medicine.

One underrepresented population that routinely utilizes fertility care is women without male partners, including lesbian couples and single women. Often, the first-line fertility treatment pursued is intrauterine insemination (IUI), but there is a paucity of research examining the IUI outcomes of women without male partners. Moreover, the limited evidence is conflicting, with some studies noting improved outcomes for lesbian patients [7,8], while others did not [9,10]. Furthermore, for women without a male partner, age-related fertility decline is compounded by delays in accessing sperm. As a result, attempts at conception often occur later, increasing the risk of involuntary childlessness and smaller-than-desired families. While the option of social (planned) egg freezing is more available in the United States than in many other countries, high costs, uneven coverage, and the fact that it postpones rather than solves age-related decline mean that it only partly offsets the risks of delayed conception without a sperm partner. Thus, as utilization of fertility treatments by women without male partners increases over time, there is an imperative to broaden our knowledge of treatment outcomes for this population. To our knowledge, this is the first study to provide insight into the detailed pregnancy and neonatal outcomes of women without male partners undergoing intrauterine insemination as compared to those with male partners.

## 2. Materials and Methods

### 2.1. Participants

This study is a retrospective chart review of IUI cycles completed at the Stanford Fertility and Reproductive Medicine Center from March 2017 through March 2023. It included all monitored IUI cycles, totaling 2414 cycles. Prior to IUI, a normal uterine cavity was verified via hysterosalpingogram, saline sonogram, hysteroscopy, or transvaginal pelvic ultrasound. Demographic and clinical characteristics, fertility treatments, and pregnancy outcomes were collected from medical records. Baseline characteristics included age, race/ethnicity, height, weight, body mass index (BMI; kg/m^2^), infertility diagnosis, type of IUI, baseline antral follicle count, endometrial thickness at ovulation trigger, presence of trilaminar pattern at trigger, size of leading follicle at trigger, and semen analysis. Beta human chorionic gonadotropin (βhCG) was collected 2 weeks after an IUI procedure. The Stanford University Institutional Review Board approved the study protocol. The patient cohort in this study has been previously described in Zhang et al., 2024, which examined the impact of estradiol supplementation on endometrial thickness and IUI outcomes [11].

### 2.2. Study Outcomes

The primary outcomes were the rates of clinical pregnancy, clinical miscarriage, and live birth. Pregnancy was defined as a positive serum β-hCG (>5 mIU/mL), clinical pregnancy was defined as viable intrauterine pregnancy with fetal cardiac activity, biochemical miscarriage was defined as rise and fall in serum β-hCG level, ectopic pregnancy was defined as pregnancy located outside the uterine cavity, clinical miscarriage was defined as pregnancy loss prior to 20 weeks of gestation, and live birth was defined as live infant born on and after 24 weeks of gestation. The secondary outcomes were prevalence of obstetric complications and neonatal outcomes. Obstetric complications included: hypertensive disorders of pregnancy, placenta disorders (including placental abruption, previa, or accreta spectrum), gestational diabetes, preterm premature rupture of membranes, postpartum hemorrhage, and cesarean section. Neonatal outcomes included: prevalence of preterm birth (<37 weeks), birth weight, rate of low birth weight (<2500 g), rate of very low birth weight (<1500 g), Apgar scores at 1 and 5 min, neonatal intensive care unit (NICU) admission, congenital anomaly, and neonatal morbidity (defined by the presence of hypoglycemia, hypothermia, intraventricular hemorrhage, necrotizing enterocolitis, seizure, infection, sepsis, or respiratory distress syndrome).

### 2.3. IUI Protocol

The standard protocol for intrauterine insemination started with a baseline ultrasound evaluation on cycle days 2 to 5. Subsequently, ovulation would be monitored with home detection kits and/or clinic ultrasounds. Both natural and medicated IUI cycles were included. Medicated cycles utilized either clomiphene citrate (50–100 mg daily starting on cycle day 2–3 for 5 days), letrozole (2.5–7.5 mg daily starting on cycle days 2–3 for 5 days), or gonadotropins (150–225 IU starting on cycle day 2–3 until ovulation trigger). Regular ultrasound monitoring would be performed around 7–9 days after ovulation induction start or 9–11 days after menses (in natural cycles) until a follicle achieved at least 18 mm in size. Thereafter, ovulation was triggered with either hCG (5000 IU) or recombinant hCG (250 mcg) followed by IUI around 36 h after ovulation.

For women without male partners, sperm was obtained either from direct donors or from anonymous donors sourced from a selection of cryobanks approved by the clinic. Eligibility for donor sperm utilization was contingent upon a comprehensive assessment including evaluation of the recipient by a reproductive mental health provider, genetic screening of the recipient to compare with the donor’s carrier testing, infectious disease testing of the sperm donor (HIV, Hepatitis B surface antigen, Hepatitis C antibody, Syphilis, Human T-lymphotropic virus) testing required by the Federal Drug Administration within the preceding 2 years. Additionally, the recipient would undergo CMV testing if the donor was CMV positive. All utilization of donor sperm was coordinated by our designated Third Party team.

### 2.4. Statistical Analysis

Study data were managed on REDcap [12], and analyses were performed on SAS Version 9.4 (SAS Institute Inc., Cary, NC, USA) statistical software. Continuous variables were presented as median and interquartile range (IQR) and compared using Wilcoxon rank-sum test. Categorical variables were presented as frequencies and percentages and compared using chi-squared or Fisher’s exact test. Overall pregnancy and clinical pregnancy rates were calculated per total number of completed IUI cycles. Rates of multiple pregnancy, clinical and biochemical miscarriage were calculated per total number of pregnancies. Live birth rates were calculated as the total number of live births divided by the total number of completed IUI cycles. Cumulative pregnancy/live birth rate per cycle was calculated as the number of pregnancies/live births from the first to the current cycle divided by the initial number of patients. Generalized estimating equations (GEE) were used to assess associations between partner status and both pregnancy outcomes (clinical pregnancy, miscarriage, and live birth) and neonatal outcomes (gestational age at delivery and birthweight). Odds ratios (ORs) and 95% confidence intervals (CIs) were reported, and models were adjusted for age, BMI, race/ethnicity, total motile count, and infertility diagnosis (diminished ovarian reserve, ovulatory dysfunction, PCOS, recurrent pregnancy loss, unexplained infertility, endometriosis); the infertility diagnoses were selected given their clinically significant differences among the two cohorts and the potential independent impact on the female participant’s ability to conceive. Poisson regression was used to evaluate the association between partner status and number of pregnancies, controlling for age, BMI, race/ethnicity, and infertility diagnosis. All statistical tests were two-tailed, and *p* < 0.05 indicated statistical significance.

## 3. Results

### 3.1. Baseline Characteristics

A total of 2414 completed IUI cycles from March 2017 through March 2023 were included in the study. Women without male partners were significantly older with a median age of 42 years compared to 38 years among women with male partners (*p* < 0.0001). Both cohorts had similar median BMIs. The two cohorts had statistically different compositions of race/ethnicity and IUI type. In our cohort, women without male partners were predominantly Caucasians (50%) compared to predominantly Asians (53%) among women with male partners; the women without male partners cohort underwent more natural cycle while the reference cohort underwent more medicated cycles (Table 1). With regard to infertility diagnosis, the two cohorts had different proportion of diagnoses, which is expected as 84.1% of women without male partners did not have a diagnosis of the common causes of female infertility. With respect to cycle characteristics, both cohorts had similar baseline antral follicle count, endometrial thickness at ovulation trigger, proportion of trilaminar endometrial pattern on ultrasound, and leading follicle size at trigger. Median sperm concentrations were comparable between the groups; however, donor semen used for IUI in women without male partners exhibited significantly lower average motility and, consequently, a lower total motile sperm count compared to semen used for women undergoing IUI with partner sperm (10.4 million vs. 18.5 million, respectively; *p* < 0.0001). This difference is likely attributable to the use of cryopreserved donor sperm specimens (with often guaranteed TMC of 10 million motile sperm) versus fresh partner sperm samples.

### 3.2. IUI Outcomes

**Figure 1:**
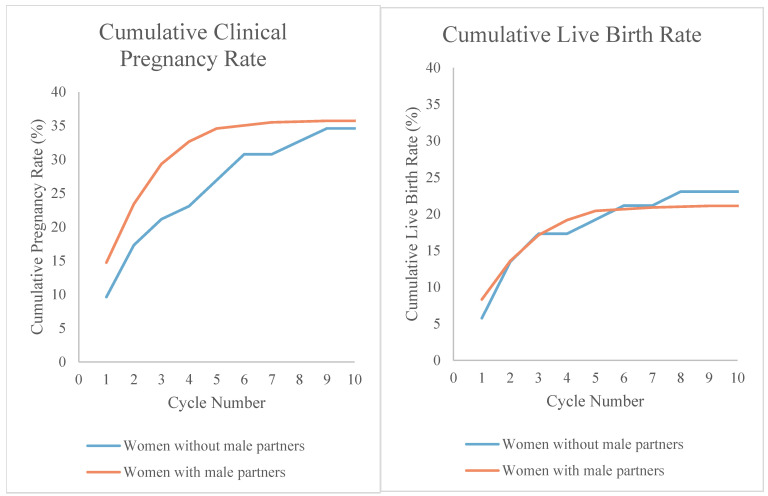
Cumulative clinical pregnancy and live birth rates by partner status.

Both cohorts completed a median of 2 cycles of IUI and underwent the same median number of IUI cycles until pregnancy and live birth (Table 2). For women without male partners, the clinical pregnancy rate per cycle was 11.4% compared to 12.5% for the reference group (*p* = 0.52), the clinical miscarriage rate was 27.8% compared to 29.8% for the reference group (*p* = 0.50), and the live birth rate was 8.1% compared to 8.2% for the reference group (*p* = 0.95). The rate of multiple pregnancy was 5.9% for women without male partners compared to 2.8% for the reference group (*p* = 0.23); all were twin pregnancies with no higher order multiples observed. The cumulative pregnancy rates were also similar between the two cohorts (Table 2 and Figure 1). When adjusted for confounders, the cumulative pregnancy rate and odds of pregnancy per cycle, miscarriage, and live birth were not significantly different (Table 3). Although statistical adjustments were made considering maternal age, the magnitude of the age difference between the two cohorts does raise concerns that residual confounding may persist.

### 3.3. Pregnancy and Neonatal Outcomes

With regard to pregnancy outcomes, women without male partners had a trend towards increased prevalence of hypertensive disorders of pregnancy (16.7% vs. 7.3%, *p* = 0.16), including preeclampsia with severe features (11.1% vs. 1.9%, *p* = 0.06) and HELLP syndrome (1% vs. 0%, *p* = 0.05). Otherwise, the two cohorts had similar rates of other hypertensive disorders of pregnancy (gestational hypertension, preeclampsia without severe features, eclampsia, and chronic hypertension with superimposed preeclampsia), placenta disorders, gestational diabetes, preterm premature rupture of membranes, postpartum hemorrhage, and cesarean section (Table 4).

With regard to neonatal outcomes, the mean and median gestational age at delivery were similar between the two cohorts (Table 4); women without male partners had neonates with a mean gestational age at delivery of 269.5 days (38 weeks and 3.5 days) compared to the reference cohort’s mean gestational age of 270.9 days (38 weeks and 4.9 days, *p* = 0.67). The median birthweights were similar (3200 g for neonates of women without male partners vs. 3140 g, *p* = 0.69). There were also no statistically significant differences in the two cohorts’ preterm birth rates, rates of low and very low birth weight, mean Apgar scores at 1 and 5 min, rates of NICU admission, neonatal morbidity, and congenital anomaly (Table 4).

## 4. Discussion

This study found that women without male partners undergoing IUI had similar rates of pregnancy, clinical miscarriage, and live birth; this persisted after adjusting for confounders such as maternal age, BMI, race/ethnicity, and infertility diagnosis (diminished ovarian reserve, ovulatory dysfunction, PCOS, recurrent pregnancy loss, unexplained infertility, endometriosis). The cohort of women without male partners median clinical pregnancy rate per cycle was 11.4% compared to 12.5% for the reference group, which is comparable to rates observed in prior IUI studies [13,14]. Both cohorts completed a median of 2 cycles of IUI and did not have statistically significant differences in cumulative pregnancy rates or in average number of IUI cycles completed to achieve pregnancy and live birth. There was a trend towards increased prevalence of hypertensive disorders of pregnancy (including preeclampsia with severe features and HELLP syndrome), but there were otherwise no significant differences in obstetric complications or neonatal outcomes such as multiple pregnancy rate, preterm birth, low birth weight, Apgar score at 1 and 5 min, NICU admission, or congenital anomaly.

There are very few studies thus far that have examined the IUI outcomes of women without male partners, and the results have been mixed. A study by Johal et al. in 2021 of lesbian women undergoing IUI found similar rates of pregnancy and live birth compared to heterosexual women but found that lesbians had higher odds of pregnancy success when adjusting for potential confounders such as maternal age, total motile sperm count, and year of procedure [8]. Their study, however, did not include single women or further examine pregnancy or neonatal outcomes. Nordqvist et al.’s 2014 study similarly compared IUI outcomes between lesbian and heterosexual women, but they observed no differences in pregnancy or live birth rates [10]. Wrande et al.’s more recent 2021 study compared lesbian women undergoing IUI to single women and observed no significant differences in live birth rates [9].

With regard to obstetric and neonatal outcomes, prior IUI studies have predominantly been in the context of women with male partners experiencing male infertility [15,16]. In a 2021 systematic review and meta-analysis examining obstetric and perinatal complications of pregnancies conceived using donor sperm compared to partner sperm, there was lower-quality evidence that use of donor sperm demonstrated a mildly increased risk of small for gestational age, but there were no differences in birth weights or other obstetric and perinatal complications [17]. Similarly to our observed trend towards higher hypertensive disorder or pregnancy risk, they did also note mild increased relative risk of hypertensive disorders of pregnancy, consistent with prior literature [17,18,19]. Diego et al.’s 2022 study examining pregnancies conceived from donor sperm included women without male partners undergoing IUI, but their study aimed to characterize prevalence of pregnancy and neonatal complications among this population [18]; thus, comparison analyses with a reference cohort were not performed. The observed trend toward increased hypertensive disorders of pregnancy in women without male partners is thought to be multifactorial, with immunologic and underlying maternal characteristics (such as more advanced maternal age) implicated. From the immunologic perspective, the lack of prior exposure to paternal antigens for women using donor sperm may impair maternal immune adaptation to the fetus, which subsequently increases the risk of abnormal placentation and preeclampsia. This is supported by studies showing higher rates of preeclampsia with donor sperm compared to partner sperm, suggesting that the antigenic novelty of donor sperm is a key factor [17,20].

To our knowledge, this is the first study examining the detailed pregnancy and neonatal outcomes of women without male partners undergoing intrauterine insemination as compared to those with male partners. It included all completed IUI cycles at our institution within the 6-year study period, thereby minimizing selection bias. All patient and cycle information was gathered from medical records to minimize recall bias. The main limitation of our study was its sample size of women without male partners given that our data was from patients within a single academic center. The small sample size of women without male partners may limit the study’s power to detect smaller, but clinically relevant, differences in obstetric complications. There were also limitations in the generalizability of our data given that our patients overall had normal BMIs in both cohorts and that the prevalence of obesity in the United States is over 40% and that obesity is one of the leading obstetric comorbidities [21,22]. Furthermore, our patient population was predominantly of Caucasian or Asian race.

In hopes of providing equitable reproductive health care regardless of marital status, sexual orientation, or gender identity, it is imperative that scientific knowledge creates a solid foundation upon which clinicians may provide personalized, evidence-based care. As more diverse patient populations increase utilization of fertility services such as IUI, there needs to be a similar growth in the body of data on which we build a better foundation of knowledge with regard to treatment outcomes. Thus, this study aims to further contribute to our understanding of outcomes for women without male partners undergoing intrauterine insemination, in hopes of minimizing the future need to infer from data based upon heterosexual couples.

## Figures and Tables

**Table 1:** Patient demographics and baseline clinical characteristics. Data are presented as median [interquartile range] or *n* (%).

	Women Without Male Partners (*n* = 149 *)	Reference: Women with Male Partners (*n* = 2265 *)	*p*-Value
**Age (years)**	42 [37, 44]	38 [35, 41]	<0.0001
**Maternal BMI (kg/m^2^)**	24.7 [21.1, 31.1]	24.2 [21.8, 27.5]	0.35
**Cycle type**			<0.0001
Clomiphene citrate	41 (27.5)	883 (39.0)	
Letrozole	42 (28.2)	919 (40.6)	
Natural cycle	49 (32.9)	270 (11.9)	
Gonadotropins	17 (11.4)	182 (8.0)	
Unknown	0 (0.0)	11 (0.5)	
**Race/Ethnicity**			<0.0001
Caucasian	26 (50.0)	236 (26.9)	
Asian American	9 (17.3)	465 (53.0)	
Hispanic/Latino	8 (15.4)	60 (6.8)	
African American	3 (5.8)	13 (1.5)	
Middle Eastern	1 (1.9)	6 (0.7)	
Other	3 (5.8)	51 (5.8)	
Unknown	2 (3.8)	47 (5.4)	
**Infertility Diagnosis**			
Male factor	0 (0.0)	162 (18.5)	0.001
Diminished ovarian reserve	7 (13.5)	229 (26.1)	0.04
PCOS	0 (0.0)	62 (7.1)	0.05
Ovulatory dysfunction	1 (1.9)	84 (9.6)	0.08
Endometriosis	0 (0.0)	30 (3.4)	0.41
Uterine factor	1 (1.9)	23 (2.6)	1.00
Tubal disease	0 (0.0)	21 (2.4)	0.63
Recurrent pregnancy loss	0 (0.0)	32 (3.6)	0.25
Sexual dysfunction	0 (0.0)	15 (1.7)	1.00
Congenital adrenal hyperplasia	0 (0.0)	1 (0.1)	1.00
Unexplained	0 (0.0)	289 (33.0)	<0.0001
**Baseline antral follicle count (n)**	9 [6, 15]	11 [5, 17]	0.12
**Cycle day at trigger**	12 [11, 13]	12 [11, 14]	0.39
**Endometrial thickness at trigger (mm)**	7.8 [7.1, 8.9]	8.0 [7.0, 9.0]	0.59
**Presence of trilaminar pattern at trigger**	102 [82.9]	1593 [79.1]	0.30
**Size of leading follicle at trigger (mm)**	19 [17, 21]	19 [17, 21]	0.55
**Sperm concentration (millions/mL)**	45.4 [32.0, 71.2]	46.3 [19.8, 100.1]	0.90
**Percent motility (%)**	50 [39, 58]	85 [70, 92]	<0.0001
**Total motile sperm count (millions)**	10.4 [6.8, 17.2]	18.5 [6.5, 45.2]	<0.0001

* Total number of IUI cycles. For demographic variables such as age, race, BMI, and infertility diagnoses, the denominator was the total number of patients in that cohort so as to not overcount patients who had undergone multiple cycles. Some patients did have multiple infertility diagnoses.

**Table 2:** Comparison of cycle and IUI outcomes for women without male partners to reference cohort of women with male partners. Data are presented as *n* (%) or median [interquartile range].

	Women Without Male Partners (*n* = 149)	Reference: Women with Male Partners (*n* = 2265)	*p*-Value
Positive βhCG	18 (12.1)	316 (14.9)	0.52
Clinical pregnancy	17 (11.4)	282 (12.5)	0.56
Biochemical miscarriage	1(5.6)	28(8.9)	0.30
Ectopic pregnancy	0 (0.0)	4 (1.3)	1.00
Clinical miscarriage	5(27.8)	94(29.8)	0.50
Live birth	12 (8.1)	186 (8.2)	0.95
Multiple pregnancy (twins)	1(5.9)	8(2.8)	0.23
Number of IUI cycles completed	2 [1, 4]	2 [1, 3]	0.05
Number of IUI cycles until pregnancy	2 [1, 5]	2 [1, 3]	0.07
Number of IUI cycles until live birth	2 [2, 4]	2 [1, 3]	0.26
Cumulative pregnancy rate:	11 (21.2)	257 (29.3)	0.21
Cycle 1	9.6	14.8	0.30
Cycle 2	17.3	23.3	0.31
Cycle 3	21.2	29.3	0.21
Cycle 4	23.1	32.6	0.15
Cycle 5	26.9	34.6	0.26
Cumulative live birth rate:			
Cycle 1	5.8	8.3	0.79
Cycle 2	13.5	13.6	0.99
Cycle 3	17.3	17.1	0.97
Cycle 4	17.3	19.2	0.74
Cycle 5	19.2	20.4	0.84

**Table 3:** Generalized linear mixed models and Poisson regression to evaluate the association between partner status and pregnancy and birth outcomes.

Pregnancy Outcomes	Adjusted Odds Ratio (aOR)	95% Confidence Interval	*p*-Value
Pregnancy rate per cycle ^a^	1.11	0.61, 2.02	0.73
Clinical miscarriage ^b^	0.75	0.15, 3.76	0.73
Live birth ^c^	1.47	0.74, 3.09	0.31
	**Rate Ratio (RR)**	**95% Confidence Interval**	***p*-Value**
Cumulative pregnancy rate	0.96	0.60, 1.55	0.87
**Neonatal Outcomes ^d^**	**Adjusted Mean Difference**	**95% Confidence Interval**	***p*-Value**
Gestational age at delivery (days)	−0.9	−7.6, 5.9	0.80
Birthweight (grams)	148.0	−190.9, 487.0	0.39

^a^ Adjusted for the following confounders: maternal age, maternal BMI, race/ethnicity, infertility diagnosis (diminished ovarian reserve, ovulatory dysfunction, PCOS, recurrent pregnancy loss, unexplained infertility, endometriosis), and sperm motility. ^b^ Adjusted for the following: maternal age, maternal BMI, race/ethnicity, and infertility diagnosis (diminished ovarian reserve, ovulatory dysfunction, PCOS, recurrent pregnancy loss, unexplained infertility, endometriosis). ^c^ Adjusted for the following: maternal age, maternal BMI, race/ethnicity, and infertility diagnosis (diminished ovarian reserve, recurrent pregnancy loss, unexplained infertility). ^d^ Adjusted for maternal age, maternal BMI and race/ethnicity.

**Table 4:** Comparison of pregnancy and neonatal outcomes between for women with and without male partners. Data are presented as *n* (%) or median [interquartile range].

	Women Without Male Partners (*n* = 18)	Reference: Women with Male Partners (*n* = 316)	*p*-Value
**Pregnancy complications**			
Any hypertensive disorder of pregnancy	3 (16.7)	23 (7.3)	0.16
Gestational hypertension	0 (0.0)	9 (2.9)	0.27
Pre-eclampsia without severe features	0 (0.0)	7 (2.2)	0.19
Pre-eclampsia with severe features	2 (11.1)	6 (1.9)	0.06
Eclampsia	0 (0.0)	0 (0.0)	**--**
HELLP syndrome	1 (5.6)	0 (0.0)	0.05
Chronic hypertension with superimposed preeclampsia	0 (0.0)	1 (0.3)	1.00
Placenta disorder (abruption, previa, accreta spectrum)	1 (5.6)	3 (1.0)	0.20
Gestational diabetes	2 (11.1)	34 (10.8)	1.00
Preterm premature rupture of membranes	0 (0.0)	5 (1.6)	1.00
Postpartum hemorrhage	0 (0.0)	14 (4.4)	1.00
Cesarean section	4 (22.2)	56 (17.8)	0.54
**Neonatal outcomes**			
Gestational age at delivery (days)			
Mean ± SD	269.5 ± 10.3	270.9 ± 13.5	0.67
Median [IQR]	273 [262, 275]	274 [266, 279]	0.31
Preterm birth (<37 weeks)	2 (16.7)	15 (8.1)	0.27
Birth weight (grams)	3200 [2530, 3600]	3140 [2807, 3445]	0.69
Low birth weight (<2500 g)	2 (16.7)	18 (9.7)	0.35
Very low birth weight (<1500 g)	0 (0.0)	2 (1.1)	1.00
Neonate’s sex			0.79
Female	6 (50.0)	92 (49.5)	
Male	5 (41.7)	83 (44.6)	
Unknown	1 (8.3)	11 (5.9)	
Apgar score at 1 min	8 [8, 9]	8 [8, 9]	0.25
Apgar score at 5 min	9 [9, 9]	9 [9, 9]	0.94
NICU admissions	0 (0.0)	20 (10.8)	0.61
Neonatal morbidity	0 (0.0)	6 (3.2)	1.00
Congenital anomaly	0 (0.0)	8 (4.3)	1.00

## Data Availability

The original data presented in the study are openly available in Dryad at 10.5061/dryad.3n5tb2rx9.
